# The Effects of Lipid Extracts from Microalgae *Chlorococcum amblystomatis* and *Nannochloropsis oceanica* on the Proteome of 3D-Cultured Fibroblasts Exposed to UVA Radiation

**DOI:** 10.3390/antiox14050545

**Published:** 2025-04-30

**Authors:** Sinemyiz Atalay Ekiner, Agnieszka Gęgotek, Maria Rosário Domingues, Pedro Domingues, Elżbieta Skrzydlewska

**Affiliations:** 1 Department of Analytical Chemistry, Medical University of Bialystok, Mickiewicza 2D, 15-222 Bialystok, Poland; agnieszka.gegotek@umb.edu.pl (A.G.); elzbieta.skrzydlewska@umb.edu.pl (E.S.); 2 Mass Spectrometry Centre, LAQV-REQUIMTE, Department of Chemistry, University of Aveiro, Santiago University Campus, 3810-193 Aveiro, Portugal; mrd@ua.pt (M.R.D.); p.domingues@ua.pt (P.D.)

**Keywords:** human skin fibroblast, UVA radiation, marine microalgae *Nannochloropsis oceanica*, freshwater microalgae *Chlorococcum amblystomatis*, redox balance, wound healing, proteomics

## Abstract

*Nannochloropsis oceanica* and *Chlorococcum amblystomatis* exhibit significant potential for protecting skin cells from oxidative stress-induced metabolic dysfunctions, owing to their high bioactive lipid content. This study aimed to evaluate their cytoprotective effects on the ultraviolet A (UVA)-perturbed proteome of 3D-cultured skin fibroblasts, using high-throughput proteomics. *Chlorococcum amblystomatis* lipid extract promoted a reduction in UVA-induced cytochrome c oxidase subunit 4 isoform 1 and cell death protein 6 levels, alongside the restoration of ferritin light chain expression diminished by UVA. It downregulated the expression of ubiquitin-conjugating enzyme E2 and lactoylglutathione lyase, which were upregulated by UVA. Furthermore, the elevated superoxide dismutase [Mn] mitochondrial levels in the caspase-1 interactome emphasized the lipid extract’s role in mitigating oxidative stress-associated chronic inflammation by regulating caspase-1 activity. In addition to this notable redox balance-regulating and cytoprotective activity, conversely, the protein inflammation signaling mediated by UVA was regulated in terms of wound healing potential in the case of *Nannochloropsis oceanica* lipid extract. Following UVA radiation, it promoted the upregulation of complement component B, thrombospondin-1, MMP1, and fibulin-1. The results revealed that both lipid extracts effectively reversed the UVA-perturbed proteomic profile of fibroblasts, highlighting their therapeutic potential in protecting the skin from UV radiation.

## 1. Introduction

Ultraviolet (UV) radiation, mainly UVA, penetrates the entire human skin, inducing metabolic modifications in both the epidermis and dermis. The observed metabolic effects are a response to the increased production of ROS and RNS (reactive oxygen and nitrogen species) and disturbances in the cellular capacity for antioxidant activity, which causes oxidative stress [1]. As a result, oxidative modifications of macromolecular compounds, including lipids and proteins, occur [2]. Moreover, the increased generation of lipid peroxidation products additionally promotes oxidative modifications, including proteins, changing their structure and functionality, which can lead to modulation of intracellular molecular signaling, especially in inflammatory reactions and the dynamics of cell survival/differentiation and, as a consequence, to disturbed cellular metabolism, including the development of skin diseases [2]. However, ROS also play an important role in intracellular signaling. ROS generation is also crucial for the molecular regulation of the wound healing process and signal transduction [3]. As in many other cases, a slight overproduction of ROS is beneficial, but excessive production can dysregulate cell functions, including promoting both the formation and healing of wounds [3,4], which ultimately, in combination with inflammation, can lead to carcinogenesis [5].

The marine microalga *Nannochloropsis oceanica* (*N. oceanica*) and the freshwater microalga *Chlorococcum amblystomatis* (*C. amblystomatis*) hold significant therapeutic promise for developing novel strategies targeting skin diseases accompanied by chronic inflammation, including psoriasis and atopic dermatitis [6,7]. These microalgae also show potential in combating skin cancers, including melanoma [8], where oxidative stress is a critical factor in disease initiation and progression. The literature highlights that lipid extracts from these microalgae can modulate intracellular redox balance and inflammation owing to their rich content of bioactive lipids, especially polyunsaturated fatty acids, which regulate intracellular signaling and lipid-soluble compound levels [6,9].

Lipidomic studies have demonstrated the antioxidant properties of *N. oceanica*, which is rich in glycolipids and oleic acid [10]. Similarly, the lipid extract of *C. amblystomatis* has shown considerable therapeutic potential in maintaining redox balance, attributed not only to its fatty acid content but also to carotenoids and chlorophyll [11]. Additionally, microalgal extracts have demonstrated beneficial effects on wound healing, attributed to their antioxidant and anti-inflammatory properties, including quenching reactive oxygen species (ROS), suppressing inflammatory cytokines and signaling cascades, and inhibiting microbial growth at wound sites [12].

The lipid extract from *N. oceanica* has been shown to promote anti-inflammatory and antioxidant responses by reducing pro-inflammatory arachidonic acid levels, inhibiting phospholipase A2, cyclooxygenase-1/2, and lipoxygenase-5 activities, and reducing the expression levels of CB1 (cannabinoid receptor type 1) and TRPV1 (transient receptor potential cation channel subfamily V member 1) receptors [13]. Concurrently, it increased polyunsaturated fatty acids, including docosahexaenoic acid and eicosapentaenoic acid, in UVB-irradiated keratinocytes [13]. In skin fibroblasts, *N. oceanica* reduced UVB-induced metabolic disturbances by lowering the levels of TNFα (tumor necrosis factor-alpha), isoprostane 8-iso PGF 2α (8-iso-prostaglandin F2), as well as the formation of protein adducts with 4-HNE (4-hydroxynonenal) [14]. Additionally, it decreased ROS levels and enhanced the activity of antioxidant enzymes, such as Cu,Zn-SOD and Mn-SOD (superoxide dismutase 1 and 2) and CAT (catalase) [14].

*C. amblystomatis* lipid extracts have also demonstrated promising therapeutic effects by significantly reducing neutral lipid accumulation in fatty-acid-overloaded liver cells [15]. In UVA-irradiated skin fibroblasts, lipid extracts from both *N. oceanica* and *C. amblystomatis* exhibited significant antioxidant properties as well as anti-inflammatory effects [16]. These effects involved reducing UVA-induced levels of endocannabinoids and eicosanoids (prostaglandin E2, thromboxane B2, and 15-hydroxyeicosatetraenoic acid), inhibiting ROS production, and stimulating Nrf2 (nuclear factor erythroid 2-related factor 2) expression [16].

Our previous study, using traditional 2D cell culture, showed the regenerative effects of the currently analyzed algal lipid extracts on the proteome changes including redox balance and regulation of inflammatory pathways, in relation to UVA-induced changes [17]. To better assess algal extracts’ effects on the proteome of skin fibroblasts, a 3D cell culture model was used in this study.

In this study, we utilized a 3D cell culture model, which offers superior insights into cellular biology compared to traditional 2D models [18], to investigate the cytoprotective effects of these lipid extracts on fibroblast metabolism disrupted by UVA-induced oxidative stress [19]. Fibroblasts, the primary cells of the dermal layer of the skin [20], were analyzed at the proteomic level. Given the strong interplay between oxidative stress and inflammation [21], we further investigated the caspase-1 protein environment, emphasizing its interaction with inflammasome complexes, playing a central role in driving inflammatory responses [22].

## 2. Materials and Methods

### 2.1. Microalgae

Spray-dried (powder form) *Nannochloropsis oceanica* (marine) and *Chlorococcum amblystomatis* (freshwater) were delivered by Allmicroalgae (located in Rua 25 de Abril s/n 2445-413 Pataias, Portugal) [23]. *N. oceanica* and *C. amblystomatis* were cultivated using Guillard’s F2 medium, adapted with locally sourced water and supplemented with magnesium and NaCl with a salinity of 30 g/L. For a duration of 7 to 15 days, they were grown in 5 L flask bioreactors, maintained under light exposure, continuously (700 µmol photons × m^2^ × s^−1^). Five 5 L flask reactors were used to inoculate a single outdoor flat panel reactor with a capacity of approximately 0.1 m^3^L that will be later expanded to 1 m^3^. Four flat panels were used in a 10 m^3^ tubular photobioreactor as inoculum. Until the stationary phase was reached, this photobioreactor was subjected to ambient light and temperature conditions, with temperature regulation achieved through a sprinkler-style irrigation system to prevent exceeding the maximum limit. pH stability was maintained by pulsed CO2 injections. When the microalgal cultures approached a concentration of 50 g/L, drying was carried out through atomization (in a spray dryer). Evaporation occurred with an efficiency of 150 kg of water per hour. The microalgal product was subjected to rapid drying in an airstream maintained at 215 ± 5 °C. The temperature of outlet air, along with biomass powder, was 92 ± 3 °C. Using a cyclone, which was protected from light and moisture, the obtained powder was stored in a place. Finally, spray-dried microalgae biomasses of *N. oceanica* and *C. amblystomatis* were utilized for the lipid extraction process.

### 2.2. Lipid Extraction Process

Lipid extraction was carried out from the spray-dried *C. amblystomatis* and *N. oceanica* biomasses via a previously described modified Folch protocol [10,24]. Briefly, the extraction process was carried out using a solvent mixture of dichloromethane–methanol (2:1, *v*/*v*) that was added to 25 mg of biomass. Following sample centrifugation at 670× *g* for 10 min, the supernatant was subjected to three additional extractions. Afterward, the supernatants (combined) were then subjected to drying under a nitrogen stream. They were then dissolved in a mixture of dichloromethane–methanol and thoroughly centrifuged. MilliQ water was added, centrifugated (670× *g* for 10 min), and the organic phase was then collected. During this time, two further extractions were performed on the aqueous phase. The obtained lipid extract comprised the entirety of the combined organic phases. Lipid quantification in the extract was performed gravimetrically. Liquid chromatography coupled with high-resolution mass spectrometry (HILIC-MS) and tandem MS (MS/MS) using a Q-Exactive hybrid quadrupole Orbitrap mass spectrometer (Thermo Fisher Scientific, Bremen, Germany) was used for the characterization of the lipid profiles of obtained extracts, as documented earlier [10].

### 2.3. Three-Dimensional (3D) Cell Culture and the Preparation of Experimental Fibroblasts Groups

The AlgiMatrix 3D (Thermo Fisher Scientific, Waltham, MA, USA) system was used to prepare the experimental human skin fibroblast cell groups. The CRL-1474 fibroblasts were provided by the American Type Culture Collection, ATCC (Manassa, VA, USA). As the first step, fibroblasts were cultured in a typical two-dimensional setup, with Dulbecco’s Modified Eagle’s Medium (DMEM) enriched with penicillin (50 U/mL), fetal bovine serum (10%), and streptomycin (50 μg/mL), at 37 °C with 5% CO_2_ in a humidified atmosphere. Once the fibroblasts achieved 90% confluence (passage no. 9), cells (5 × 10^5^ per well) were plated in 24-well plates with AlgiMatrix gel (Life Technologies, Carlsbad, CA, USA) to establish a 3D cell culture model. Then, they were cultured with standardized conditions in a growing medium (DMEM supplemented with 10% FBS, 50 μg/mL streptomycin, and 50 U/mL penicillin) for four days, according to the producer’s procedure [25]. Experimental fibroblast groups were prepared as follows:

**CTR** ► Control group, the fibroblasts which were cultured in the 3D system with standard medium;

***N.o.*** ► The fibroblasts which were cultured in the 3D system with the standardized growth medium also containing *N. oceanica* lipid extract (3 µg/mL) for a 24 h period;

***C.a.*** ► The fibroblasts which were cultured in the 3D system with the standardized growth medium also containing *C. amblystomatis* lipid extract (3 µg/mL) for a 24 h period;

**UVA** ► The fibroblasts exposed to UVA (365 nm—at a dose of 13 J/cm^3^), which were then incubated in the 3D system with the standardized growth medium for a 24 h period;

**UVA + *N.o.*** ► The fibroblasts exposed to UVA (365 nm—at a dose of 13 J/cm^3^), which were then incubated in the 3D system with the standardized growth medium containing *N. oceanica* lipid extract (3 µg/mL) for a 24 h period;

**UVA + *C.a.*** ► The fibroblasts exposed to UVA (365 nm—at a dose of 13 J/cm^3^), which were then incubated in the 3D system with the standardized growth medium containing *C. amblystomatis* lipid extract (3 µg/mL) for a 24 h period.

The concentration of lipid extracts from *N. oceanica* and *C. amblystomatis* (3 µg/mL) used for treatment and the dose of used UVA exposure at 13 J/cm^3^ were chosen based on 70% viability, using MTT assay (3-[4,5-dimethylthiazol-2-yl]-2,5 diphenyl tetrazolium bromide) assay [26] in 3D cell culture system. Thus, the selected concentration of the mentioned lipid extracts was not toxic for the skin fibroblasts cultured with the 3D system. Upon preparing the experimental fibroblast groups as mentioned above, they were recovered from 3D gel using AlgiMatrix™ dissolving buffer. The cell pellet was resuspended in tris-buffered saline (pH 8.0) with a protease inhibitor cocktail (Sigma-Aldrich, P8340) after centrifugation (300× *g*, 3 min). The cells were then lysed by sonication on ice and centrifuged (12,000× *g*, 15 min) for further analysis. The total protein concentration in the samples was quantified using the Bradford assay [27].

### 2.4. The Process of Immunoprecipitation Against Human Caspase-1 (IP Against hCasp1)

The protein–protein interaction area of human caspase-1 was analyzed through an approach that combined immunoprecipitation (IP) against caspase-1 and high-performance liquid chromatography coupled with tandem mass spectrometry (HPLC-MS/MS). The samples, containing 100 µg proteins, were pre-cleared using protein A agarose to remove molecules that could interact non-specifically with protein A agarose. By centrifugation (10,000× *g*, 1 min, 4 °C), protein A agarose was removed. Following that, the samples were incubated with anti-human Caspase-1 primary antibody (Abcam, Anti-Caspase1, ab1872) for one hour at a temperature of 4 °C. Following this step, protein A agarose was added and incubated (overnight) for precipitating the proteins bound with antibodies. Thereafter, incubated samples were centrifuged at 10,000× *g* for 10 min at 4 °C. Next, the obtained pellet (from proteins immunoprecipitated with Caspase-1) was combined with sample loading buffer (Laemmli buffer, supplemented with 5% 2-mercaptoethanol), for the next SDS-PAGE and following in-gel digestion step regarding down-stream proteomic analysis.

### 2.5. Proteomic Analysis

In addition to the final IP samples, prepared by mixing with Laemmli buffer as mentioned above, samples containing 30 μg of protein were added to sample loading buffer at a volume ratio of 1:2 and heated at 100 °C for 7 min before separation on Tris-Glycine SDS-PAGE gels (10%). Following SDS-PAGE run, the gels were fixed in methanol–acetic acid–water (at a volume ratio of 4:1:5; for a duration of 1 h) and stained using Coomassie Brilliant Blue R-250 (overnight). Entire lanes were cut from the gels and sliced into eight (complete proteomic analysis) or three (IP-adjusted proteomic analysis) sections (Supplementary Figure S1). Protein reduction and alkylation in each slice were carried out by incubating with 1,4-dithiothreitol (DTT; 10 mM) and iodoacetamide (IAA; 50 mM), respectively. After these steps, they were subjected to trypsinization (Promega, Madison, WI, USA) at 37 °C, overnight, to carry out the in-gel protein digestion process. Finally, the obtained peptide mixtures (both for the complete proteomic analysis samples and IP-adjusted proteomic analysis samples) were extracted from the gel, and the dried peptide mixtures dissolved in acetonitrile (ACN, 5%) with formic acid (FA, 0.1%).

Using Ultimate 3000 high-performance liquid chromatography (HPLC) system (Dionex, Idstein, Germany) on a 150 mm × 0.075 mm PepMap RSLC capillary analytical C18 column with 2 μm particle size (Dionex, LC Packings), the peptide mixtures were subjected to separation at a flow rate of 0.300 μL/min. For sample mobilization, Eluent A (5% ACN + 0.1% FA) and Eluent B (90% ACN + 0.03% FA) were utilized through the column, with a time gradient starting at 3 min and progressing to 60% Eluent B over a 40 min period. The peptides were examined with a Q Exactive HF mass spectrometer (positive ion calibration and in data-dependent mode) together with an electrospray ionization source (ESI) (Thermo Fisher Scientific, Bremen, Germany). The comprehensive layout regarding the HPLC-MS/MS analysis has been described in detail previously [28].

For analysis of the raw data generated by HPLC-MS/MS, the MaxQuant software (version 2.5.2.0, using andromeda search engine) [29] was used. The default parameters were used for protein identification. Protein label-free quantification (LFQ) was performed for the semi-quantitative analysis, according to the signal intensities of precursor ions. And the data were searched against the UniProtKB-SwissProt database (taxonomy: *Homo sapiens, up to date 1 July 2024*).

### 2.6. Protein Level Analysis

#### 2.6.1. Western Blotting

To confirm the MS/MS results, the expression levels of selected proteins, which are chosen from those showing the most significant changes, were analyzed using Western blotting [30]. The separated proteins (as explained in Section 2.5, electrophoresis was performed) were transferred to the nitrocellulose membranes and blocked using 5% skim milk. After blocking, the membranes were incubated with the primary antibodies against β-actin (Sigma-Aldrich, St. Louis, MO, USA, host: mouse), peroxiredoxin 5 (Sigma-Aldrich, St. Louis, MO, USA, host: rabbit), heat shock 10 kDa protein 1 (Sigma-Aldrich, St. Louis, MO, USA, host: rabbit), matrix metalloproteinases 1 (Sigma-Aldrich, St. Louis, MO, USA, host: rabbit), ferritin light chain (Sigma-Aldrich, St. Louis, MO, USA, host:rabbit), fibulin 1 (Abcam, Cambridge, UK, host: mouse), cyctochrome C (Santa Cruz Biotechnology, Santa Cruz, CA, USA, host: mouse), and RAS (Sigma-Aldrich, St. Louis, MO, USA, host: rabbit) at a concentration of 1:1000. Once primary antibody incubation was completed, the membranes were washed with TRIS-buffered saline, with Tween 20. Then, the membranes were incubated with polyclonal alkaline phosphatase secondary antibodies against mouse/rabbit (Sigma-Aldrich, St. Louis, MO, USA) for 2 h. Protein bands were visualized using the BCIP/NBT liquid substrate system (Sigma-Aldrich; St. Louis, MO, USA) and VersaDoc System-QuantityOne software (Bio-Rad Laboratories Inc., Hercules, CA, USA).

#### 2.6.2. Elisa Method

The levels of phosphorylated-Nrf2 (pNrf2, active) and heme oxygenase-1 (HO-1) were measured through the ELISA method. The samples were added to ELISA plate wells (Nunc Immuno Maxisorp, Thermo Scientific, Waltham, MA, USA) and incubated overnight at 4 °C with a pNrf2 (Bioss Antibodies, Woburn, MA, USA; host: rabbit) or HO-1 (Sigma-Aldrich, St. Louis, MO, USA, host: rabbit). The plates were incubated at room temperature for 30 min with a peroxidase blocking solution composed of 3% hydrogen peroxide and 3% skim milk, following the washing step. Goat anti-rabbit secondary antibody solution (Dako, Carpinteria, CA, USA) was added to each well and incubated for 1 h. Chromogen substrate solution (0.1 mg/mL TMB and 0.012% H_2_O_2_) was then applied to each well and allowed to incubate for 40 min. After stopping the reaction with 2 M sulfuric acid, the absorbance was measured at 450 nm (using a reference filter set at 620 nm). Finally, the levels of pNrf2 and HO-1 (normalized according to the protein concentrations, ng/mg protein) were expressed as a percentage of the expression observed in the control cells.

### 2.7. Lipid Peroxidation Product (4-HNE) Level Determination by GC-MS

The level of 4-HNE, a marker of oxidative stress, was measured by gas chromatography coupled with mass spectrometry (GC-MS) in selected ion monitoring mode (SIM), following the Tsikas method [31] with slight modifications as previously reported [32], and using 4-hydroxynonenal-d3 as the internal standard (ISTD) [16]. Initially, the ISTD and PFBHA·HCl derivatization reagent were added to the sample and incubated on a shaker at room temperature for 24 h. After the derivatization process was interrupted by methanol addition, the aldehydes were extracted into hexane. The samples were centrifuged, and the hexane layer was collected into glass tubes with a modified, chemically inactive surface. After re-extraction of the samples, the hexane layers were combined and evaporated (until dry). The (dry) residue was dissolved in a portion of the second derivatization reagent (BSTFA:TMCS) and then incubated at 80 °C for 15 min. Following mixing and cooling, the samples were transferred to chromatographic vials with glass inserts with a chemically inactive surface and a modified surface. After that, O-PFB-oxime-TMS derivatives of both 4-HNE and ISTD were analyzed through 7890A GC—7000 quadrupole MS/MS (Agilent Technologies, Palo Alto, CA, USA) which is equipped with a 30 m length HP-5MS capillary column (0.25 mm internal diameter). The content of 4-HNE was analyzed by detecting ions at *m*/*z* 242.0 for 4-HNE-PFB-TMS and *m*/*z* 245.0 for the ISTD derivative. The 4-HNE values were normalized according to the protein concentrations in the samples, with the final units reported as nmol/mg protein.

### 2.8. Wound Healing Assay (In Vitro Scratch Analysis)

Cell monolayers were scratched with a sterile pipette tip, then washed with medium to eliminate any loose cells. Scratches were then exposed to UVA (13 J/cm^3^) and/or treated with lipid extracts from *N. oceanica* and *C. amblystomatis* (3 µg/mL). Following 24 h incubation, the width of the furrow was visualized using NikonEclipse Ti microscope combined with MagingSource camera operated by Nikon’s NIS-Elements imaging software version 5.30.02 (Nikon Instruments Inc., Melville, NY, USA).

### 2.9. Statistical Analysis

Samples obtained from each experimental fibroblast group (**CTR**, ***N.o.***, ***C.a.***, **UVA**, UVA + ***N.o.***, UVA + ***C.a.***, as described above) were examined in three independent experiments. Proteins identified by MaxQuant software analysis with a minimum of two unique peptides and a q-value ≤ 0.005 (FDR confidence of 1% for both peptides and proteins) were subjected to statistical analysis. LFQ-generated protein intensities (individual) were log-transformed and normalized by median (complete proteomic analysis results in case of *N.oceanica* lipid extract treatment; IP-adjusted proteomic analysis results in case of *C.amblystomatis* lipid extract treatment) or by sum (complete proteomic analysis in the case of *C.amblystomatis* lipid extract treatment; IP-adjusted proteomic analysis in the case of *N. oceanica* lipid extract treatment) using open-source software MetaboAnalyst (version 6.0) [33]. Three-dimensional cell culture approaches, rather than traditional bidimensional in vitro cell systems, allow a good achievement of cell differentiation and complexity (compared to 2D cell models) and present a much better cell morphology, behaviors, and topology by reflecting in vivo conditions [18]. However, due to the biological variations and complexity in 3D experiments, like in vivo studies, 3D models also bring statistical concerns and challenges [34,35,36]. In the proteomic data of this study (including both complete and IP-adjusted proteomic analysis), due to the statistical concerns (mentioned before) in the normalization stage, the data were analyzed and interpreted separately (to better reflect a normal distribution) for each microalgae case. Statistical assessments, including one-way ANOVA, principal component analysis (PCA), heatmaps, and volcano plot assessments, were performed using MetaboAnalyst. The one-way ANOVA *p*-value (FDR) cut-off <0.05 was concluded to be statistically significant (together with post hoc Fisher’s test). Euclidean distance measure was used in heatmap generation. STRING (version 12.0) was used for protein annotations [37]. GO (Gene ontology) enrichment analysis was performed using the PANTHER classification system (version 19.0, utilizing Fisher’s Exact test and calculating FDR) [38,39]. The obtained GO terms (biological processes) were visualized as a pie chart (based on their fold enrichment) using ‘ggplot2’ [40] and ‘dplyr’ [41] in RStudio (version 2023.12.1 [42]).

## 3. Results

In the complete proteomic analysis and IP (hCasp1)-adjusted proteomic analysis of 3D-cultured fibroblasts, a total of 283 and 44 proteins were identified, respectively. The complete list of identified proteins, along with their names, UniProt IDs, peptide counts (razor + unique and unique), average intensities, and sequence coverage (razor + unique and unique), is available in Supplementary Table S1. One-way ANOVA revealed that treatment with *N. oceanica* lipid extracts resulted in significantly altered protein intensities for 51 proteins, while treatment with *C. amblystomatis* lipid extracts affected 49 proteins within the complete proteomic analysis of fibroblast groups (Supplementary Table S1). Conversely, within the IP (hCasp1)-adjusted proteomic analysis, only three proteins showed significantly altered intensities in the *N. oceanica* lipid extract treatment group, and one protein exhibited significant changes in the *C. amblystomatis* lipid extract treatment group (Supplementary Table S1).

**Figure 1:**
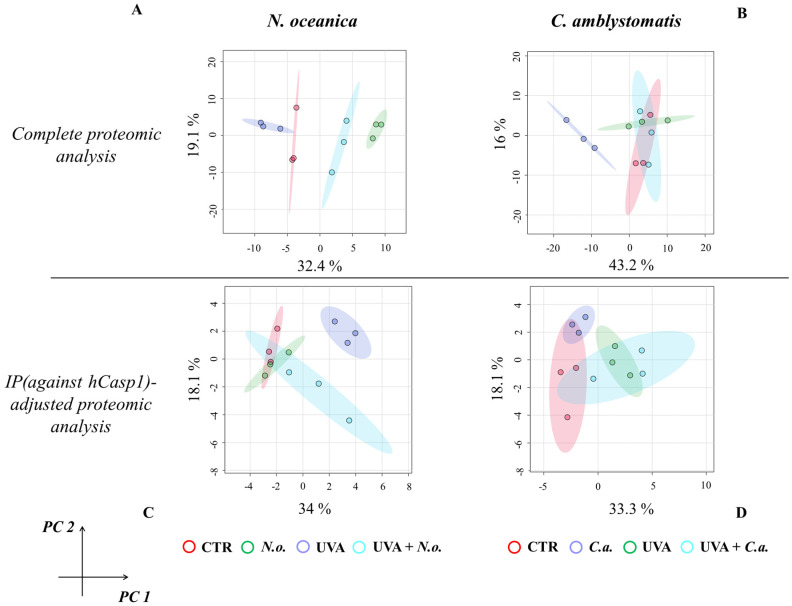
Principal component analysis of the fibroblast proteome from experimental cell groups treated with *N. oceanica* or *C. amblystomatis* lipid extracts. The analysis was conducted for the complete proteomic dataset (**A**,**B**) and the IP (hCasp1)-adjusted proteomic dataset (**C**,**D**) [**CTR**, control cells cultured in the 3D system with standard medium; ***N.o.***, the cells treated with lipid extract obtained from *N. oceanica* (3 µg/mL) for 24 h; ***C.a.***, the cells treated with lipid extract obtained from *C. amblystomatis* (3 µg/mL) for 24 h; **UVA**, the cells exposed to UVA (365 nm) at a dose of 13 J/cm^3^ and incubated in the 3D system with the standard growing medium for 24 h; **UVA + *N.o*.**, the cells exposed to UVA (365 nm) at a dose of 13 J/cm^3^ and then incubated in the 3D system with the standard growing medium containing lipid extracts from *N. oceanica* (3 µg/mL) for 24 h; **UVA + *C.a.***, the cells exposed to UVA (365 nm) at a dose of 13 J/cm^3^ and then incubated in the 3D system with the standard growing medium containing lipid extracts from *C. amblystomatis* (3 µg/mL) for 24 h; IP (against hCasp1), immunoprecipitation against human caspase−1; PC1/2, principal component 1/2].

The principal component analysis (PCA) of proteomic data obtained from experimental cell groups treated with *N. oceanica* lipid extracts in the complete proteomic analysis revealed distinct clustering. All experimental cell groups were separated into discrete clusters in the case of *N. oceanica* treatment (principal component 1—32.4%; principal component 2—19.1%; Figure 1A). In contrast, while *C. amblystomatis* lipid extract treatment clearly distinguished the *C.a.* cell group from other groups, the differentiation among the CTR, UVA, and UVA + *C.a.* cell groups was less pronounced (principal component 1—43.2%; principal component 2—16%; Figure 1B).

For the IP (hCasp1)-adjusted proteomic analysis, UVA irradiation caused the UVA cell group to distinctly separate from all other groups. However, treatment with *N. oceanica* lipid extracts following UVA exposure shifted the UVA + *N.o.* cell group closer to the CTR and *C.a.* cell groups (principal component 1—34%; principal component 2—18.1%; Figure 1C). Lastly, while the individual samples within the experimental groups did not overlap, the clustering of the experimental groups treated with *C. amblystomatis* lipid extracts was less defined (principal component 1—33.3%; principal component 2—18.1%; Figure 1D).

**Figure 2:**
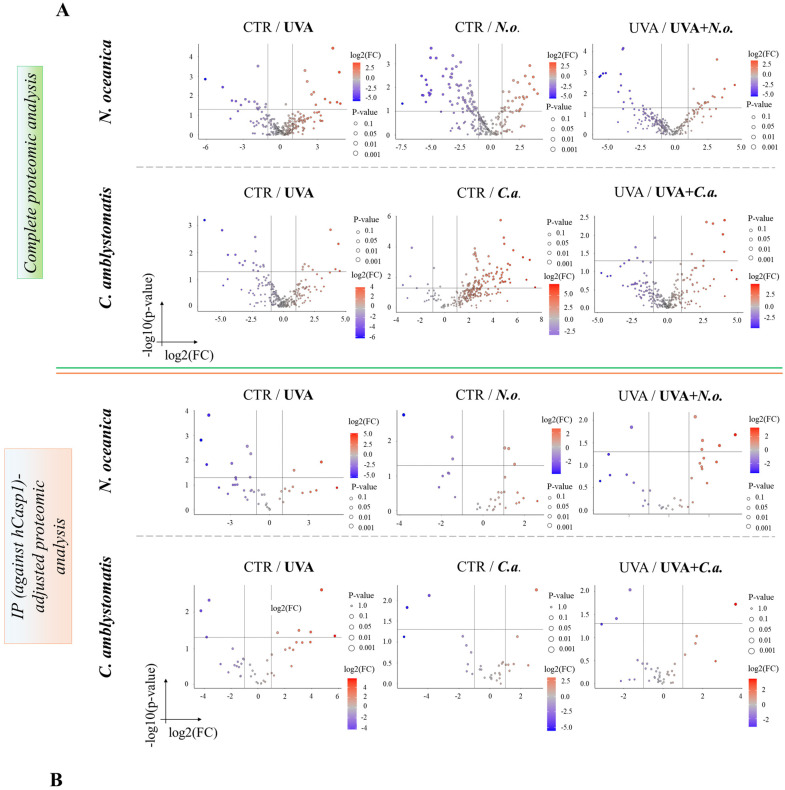
Volcano plots showing the effects of *N. oceanica* and *C. amblystomatis* lipid extracts on the proteome of fibroblasts in 3D-cultured experimental cell groups. The plots compare differential protein expression within the complete proteomic analysis (**A**) and the IP (hCasp1)-adjusted proteomic analysis (**B**) (**CTR**, control cells cultured in the 3D system with standard medium; ***N.o.***, the cells treated with lipid extract obtained from *N. oceanica* (3 µg/mL) for 24 h; ***C.a.***, the cells treated with lipid extract obtained from *C. amblystomatis* (3 µg/mL) for 24 h; **UVA**, the cells exposed to UVA (365 nm) at a dose of 13 J/cm^3^ and incubated in the 3D system with the standard growing medium for 24 h; **UVA + *N.o.***, the cells exposed to UVA (365 nm) at a dose of 13 J/cm^3^ and then incubated in the 3D system with the standard growing medium containing lipid extracts from *N. oceanica* (3 µg/mL) for 24 h; **UVA + *C.a.***, the cells exposed to UVA (365 nm) at a dose of 13 J/cm^3^ and then incubated in the 3D system with the standard growing medium containing lipid extracts from *C. amblystomatis* (3 µg/mL) for 24 h; IP (against hCasp1), immunoprecipitation against human caspase-1; significant features highlighted as blue and red as seen in figure-fold change had *p* < 0.05).

The volcano plots (Figure 2A,B) revealed significant proteomic differences in fibroblasts, both in the complete and IP-adjusted proteomic analyses, when comparing the effects of *N. oceanica* and *C. amblystomatis* lipid extracts on control fibroblasts and UVA-exposed fibroblasts. In the complete proteomic analysis, treatment with *N. oceanica* or *C. amblystomatis* lipid extracts (in the absence of UVA-induced stress) significantly altered the proteome of control fibroblasts (Figure 2A).

**Figure 3:**
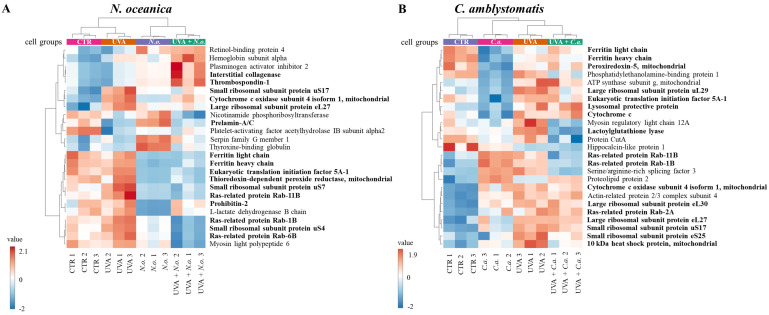
Heatmap of the top 25 proteins with significantly altered expression in fibroblasts derived from experimental cell groups treated with *N. oceanica* or *C. amblystomatis* lipid extracts (**A**,**B**) within the complete proteomic analysis. The hierarchical clustering demonstrates distinct proteomic profiles across treatment conditions (**CTR**, control cells cultured in the 3D system with standard medium; ***N.o.***, the cells treated with lipid extract obtained from *N. oceanica* (3 µg/mL) for 24 h; ***C.a.***, the cells treated with lipid extract obtained from *C. amblystomatis* (3 µg/mL) for 24 h; **UVA**, the cells exposed to UVA (365 nm) at a dose of 13 J/cm^3^ and incubated in the 3D system with the standard growing medium for 24 h; **UVA + *N.o.***, the cells exposed to UVA (365 nm) at a dose of 13 J/cm^3^ and then incubated in the 3D system with the standard growing medium containing lipid extracts from *N. oceanica* (3 µg/mL) for 24 h; **UVA + *C.a.***, the cells exposed to UVA (365 nm) at a dose of 13 J/cm^3^ and then incubated in the 3D system with the standard growing medium containing lipid extracts from *C. amblystomatis* (3 µg/mL) for 24 h). Proteins written in bold type characters present the proteins that are critical in regulating the dynamic oxidative stress and related inflammatory response as well as cell survival. Turning to the case of *C. amblystomatis* lipid extract within the complete proteomic analysis, the expression of lactoylglutathione lyase (Q04760) was found to be nearly 4-fold increased by UVA exposure but was significantly reduced following treatment with the *C. amblystomatis* lipid extract. Similarly, the UVA-induced upregulation of ubiquitin-conjugating enzyme E2 variant 1 (Q13404) was also significantly reduced by *C. amblystomatis* lipid extract treatment (approximately 0.7-fold change). On the other hand, the expression of prostaglandin E synthase 3 (Q15185) was moderately increased by UVA exposure (1.16-fold) and was further elevated slightly by *C. amblystomatis* lipid extract treatment (1.01-fold change). Additionally, the increase in eukaryotic translation initiation factor 5A-1 (P63241) expression caused by UVA exposure was significantly decreased following *C. amblystomatis* lipid extract treatment (0.7-fold change). Although this trend was also observed with *N. oceanica* lipid extract, the effect of *N. oceanica* was milder compared to *C. amblystomatis*. Furthermore, UVA exposure caused a dramatic decrease in cathepsin B (P07858) levels (0.8-fold), but *C. amblystomatis* lipid extract treatment mitigated this decline (0.05-fold change).

A heatmap of the top 25 proteins with significantly altered expression in skin fibroblasts (from the complete proteomic analysis) was generated. Due to the limited number of significantly altered proteins identified in the IP-adjusted analysis, a separate heatmap was not created. The upper hierarchical dendrogram revealed distinct clustering of samples in both *N. oceanica* and *C. amblystomatis* treatment groups, organized as follows: control cell group (CTR), lipid extract-treated groups (*N.o.*/*C.a.*), UVA-exposed group (UVA), and UVA-exposed groups treated with lipid extracts (UVA + *N.o.*/UVA + *C.a.*) (Figure 3A,B). Notably, in the *C. amblystomatis* treatment, CTR and UVA groups clustered closely, separating from the *N.o.* and UVA + *N.o.* groups (Figure 3A). In contrast, in the *N. oceanica* treatment, the CTR group was distinctly separated from the UVA, *C.a.*, and UVA + *C.a.* groups (Figure 3B).

**Figure 4:**
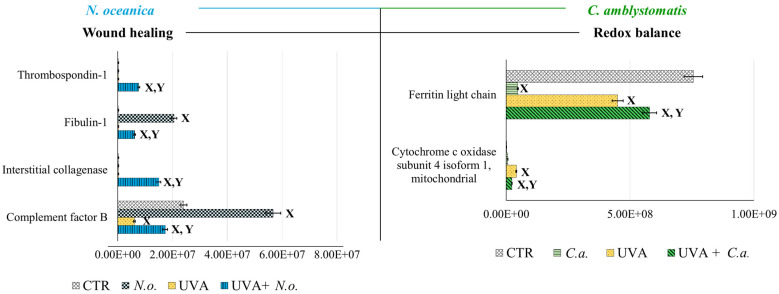
Changes in the average intensity levels of proteins involved in the regulation of wound healing (*N. oceanica*) or redox balance (*C. amblystomatis*) with significantly altered expression in fibroblasts derived from experimental cell groups (**CTR**, control cells cultured in the 3D system with standard medium; ***N.o.***, the cells treated with lipid extract obtained from *N. oceanica* (3 µg/mL) for 24 h; ***C.a.***, the cells treated with lipid extract obtained from *C. amblystomatis* (3 µg/mL) for 24 h; **UVA**, the cells exposed to UVA (365 nm) at a dose of 13 J/cm^3^ and incubated in the 3D system with the standard growing medium for 24 h; **UVA + *N.o.***, the cells exposed to UVA (365 nm) at a dose of 13 J/cm^3^ and then incubated in the 3D system with the standard growing medium containing lipid extracts from *N. oceanica* (3 µg/mL) for 24 h; **UVA + *C.a.***, the cells exposed to UVA (365 nm) at a dose of 13 J/cm^3^ and then incubated in the 3D system with the standard growing medium containing lipid extracts *from C. amblystomatis* (3 µg/mL) for 24 h). Mean values ± SD of three independent samples and statistically significant differences for *p* ≤ 0.05 are presented: (**X**) is used for differences vs. **CTR**; (**Y**) is used for differences between the group **UVA** and **UVA + *N.o.*** or **UVA + *C.a***.

According to fold-change calculations based on average protein intensities (Supplementary Table S2), significant changes in critical proteins regulating redox homeostasis were observed. In the complete proteomic analysis, cytochrome c oxidase subunit 4 isoform 1, mitochondrial (P13073), was substantially increased by UVA exposure (approximately 133-fold) but significantly reduced following *N. oceanica* treatment (0.089-fold). Similarly, *C. amblystomatis* treatment reduced the same protein’s expression (0.566-fold), exhibiting a relatively stronger effect compared to *N. oceanica* (Figure 4).

Antioxidant proteins showed varied responses: the expression of thioredoxin-dependent peroxide reductase, mitochondrial (P30048), which decreased under UVA exposure (0.751-fold), was slightly reduced further by *N. oceanica* treatment (0.056-fold). Conversely, *C. amblystomatis* treatment moderately increased the expression of another antioxidant, peroxiredoxin-5, mitochondrial (P30044), which had been reduced by UVA. Considering the relationship between iron metabolism and redox signaling, ferritin heavy chain (P02794), decreased by UVA exposure, was further reduced by *N. oceanica* (0.031-fold) and *C. amblystomatis* (0.938-fold) treatments. However, ferritin light chain (P02792), also reduced by UVA, showed a slight decrease with *N. oceanica* (0.040-fold) and a notable increase with *C. amblystomatis* treatment (~1.2-fold) (Figure 4).

Given the interplay between oxidative stress and inflammation [21,43], proteins regulating inflammation were also evaluated. Complement factor B (P00751), reduced nearly threefold by UVA, was restored to approximately threefold higher levels by *N. oceanica* treatment (Figure 4). Additionally, interstitial collagenase (matrix metalloproteinase-1, P03956), undetectable after UVA exposure, exhibited a 49-fold increase with *N. oceanica* treatment following UVA. Similar trends were observed for thrombospondin-1 (P07996), plasma serine protease inhibitor (P05120), and fibulin-1 (P23142), with respective increases of 25.16-fold, 32.7-fold, and 20.3-fold after *N. oceanica* treatment.

Importantly, *N. oceanica* lipid extract treatment significantly reduced the UVA-induced upregulation of ribosomal proteins, including small ribosomal subunit proteins uS17 (P62280), uS4 (P46781), uS7 (P46782), and large ribosomal subunit protein eL27 (P61353). Similarly, *C. amblystomatis* lipid extract treatment reduced the expression of large ribosomal subunit proteins eL24 (P83731), eL27 (P61353), eL30 (P62888), uL29 (P42766), and small ribosomal subunit proteins eS25 (P62851), uS10 (P60866), uS13 (P62269), and uS17 (P62280). *N. oceanica* lipid extract also induced a significant reduction in the expression of ras-related proteins Rab-11B (Q15907), Rab-1B (Q9H0U4), and Rab-6B (Q9NRW1), which were elevated by UVA exposure. Similarly, *C. amblystomatis* lipid extract caused a comparable reduction in Rab-11B (Q15907), Rab-1B (Q9H0U4), Rab-2A (P61019), and Rab-6B (Q9NRW1) levels.

Prohibitin-2 (Q99623) expression was markedly reduced by UVA exposure (0.79-fold). While *N. oceanica* lipid extract treatment further decreased this expression (0.29-fold), the effect was milder. *C. amblystomatis* lipid extract treatment also notably reduced the expression of programmed cell death protein 6 (O75340), which was increased by UVA exposure. Moreover, the UVA-reduced level of protein disulfide-isomerase A6 (Q15084) was increased approximately 3-fold following *C. amblystomatis* lipid extract treatment. In contrast, the UVA-induced upregulation of the 10 kDa heat shock protein, mitochondrial (P61604), was significantly reduced by *C. amblystomatis* lipid extract treatment (0.66-fold change). Similarly, calreticulin (P27797), which was lowered by UVA exposure, showed further reduction with *N. oceanica* lipid extract treatment (0.14-fold change) but to a lesser extent compared to the direct UVA effect (0.38-fold change).

**Figure 5:**
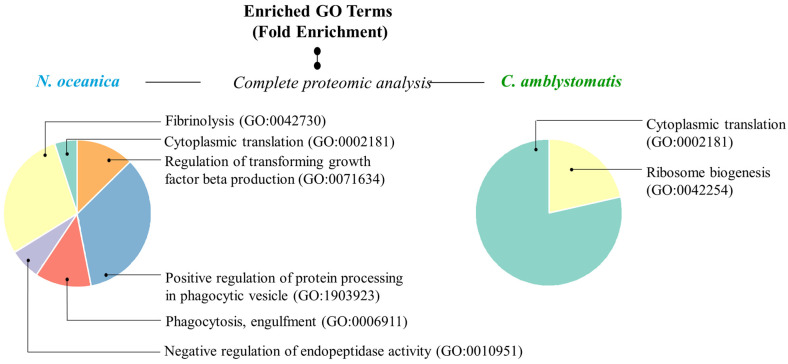
GO enrichment analysis. Pie charts displaying enriched GO terms were created using data from the PANTHER classification system, based on their fold enrichment, within the complete proteomic analysis in the case of *N. oceanica* or *C. amblystomatis* treatment of fibroblasts. Unique identifier codes assigned to specific GO terms expressing biological processes are given in parentheses. Only the proteins with statistically significant expression changes between experimental cell groups (due to the one-way ANOVA analysis) were included in the analysis.

In addition, to have a more comprehensive view of the mechanisms of action of the extracts used, Gene ontology (GO)-enrichment analysis was performed. The analysis revealed that the protein set obtained from the complete proteomic analysis of fibroblasts in the case of *N. oceanica* lipid extract was primarily associated with biological processes, specifically the positive regulation of protein processing in phagocytic vesicles (GO:1903923) and fibrinolysis (GO:0042730) (Figure 5). In contrast, the protein set associated with the *C. amblystomatis* lipid extract was strongly linked to critical biological processes, particularly cytoplasmic translation (GO:0002181) and ribosome biogenesis (GO:0042254).

**Figure 6:**
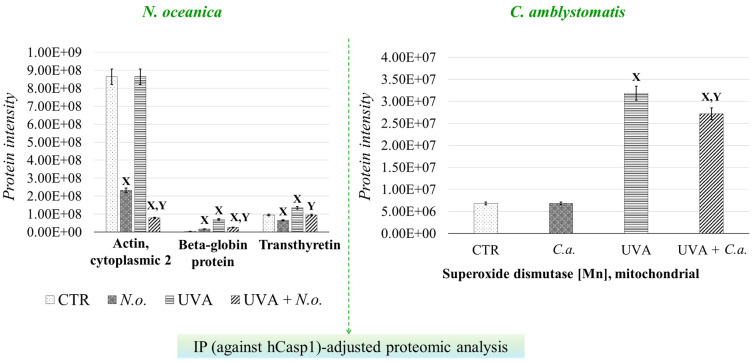
Changes in the average intensity levels of proteins with significantly altered expression in fibroblasts derived from experimental cell groups (**CTR**, control cells cultured in the 3D system with standard medium; ***N.o.***, the cells treated with lipid extract obtained from *N. oceanica* (3 µg/mL) for 24 h; ***C.a.***, the cells treated with lipid extract obtained from *C. amblystomatis* (3 µg/mL) for 24 h; **UVA**, the cells exposed to UVA (365 nm) at a dose of 13 J/cm^3^ and incubated in the 3D system with the standard growing medium for 24 h; **UVA + *N.o.***, the cells exposed to UVA (365 nm) at a dose of 13 J/cm^3^ and then incubated in the 3D system with the standard growing medium containing lipid extracts from *N. oceanica* (3 µg/mL) for 24 h; **UVA + *C.a.***, the cells exposed to UVA (365 nm) at a dose of 13 J/cm^3^ and then incubated in the 3D system with the standard growing medium containing lipid extracts *from C. amblystomatis* (3 µg/mL) for 24 h) within IP(against hCasp1)-adjusted proteomic analysis. Mean values ± SD of three independent samples and statistically significant differences for *p* ≤ 0.05 are presented: (**X**) is used for differences vs. **CTR**; (**Y**) is used for differences between the group **UVA** and **UVA + *N.o.*** or **UVA + *C.a*.** [IP (against hCasp1), immunoprecipitation against human caspase-1].

To further investigate the strong relationship between oxidative stress and inflammation, the protein interaction network of caspase-1, which interacts with inflammasome complexes—the major contributors to inflammation [22]—was analyzed as a complementary approach to the complete proteomic analysis (Supplementary Table S2, Figure 6). In the case of *N. oceanica* lipid extract treatment, the expression of beta-globin protein (Q9UM85) and transthyretin (A6XMH1) was significantly elevated by UVA exposure, showing increases of 17-fold and 1.4-fold, respectively. However, treatment with *N. oceanica* lipid extract effectively reduced these elevated levels. Additionally, while the expression level of actin, cytoplasmic 2 (P63261), remained unchanged following UVA exposure, subsequent treatment with *N. oceanica* lipid extract caused a dramatic reduction in its expression (0.09-fold change). Regarding *C. amblystomatis* lipid extract treatment, UVA exposure resulted in a remarkable 5-fold increase in the expression of superoxide dismutase [Mn], mitochondrial (P04179), in fibroblasts. Treatment with *C. amblystomatis* lipid extract significantly reduced this elevated expression caused by UVA.

**Figure 7:**
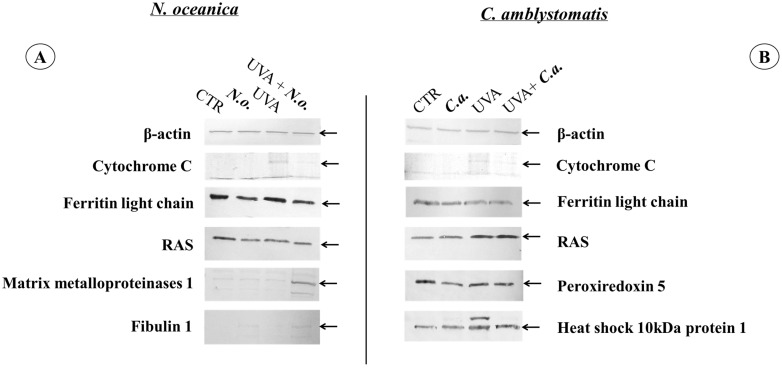
Western blot analysis against β-actin, cytochrome C, ferritin light chain, RAS, peroxiredoxin 5, heat shock 10 kDa protein 1, matrix metalloproteinases 1 or fibulin 1 in the case of *N. oceanica* (**A**) or *C. amblystomatis* (**B**) lipid extract treatment of fibroblasts [**CTR group**, control fibroblasts cultured in 3D system with standard medium; ***N.o.*** group, the fibroblasts treated with lipid extract obtained from *N. oceanica* (3 µg/mL) for 24 h; ***C.a.*** group, the fibroblasts treated with lipid extract obtained from *C. amblystomatis* (3 µg/mL) for 24 h; **UVA** group, the fibroblasts exposed to UVA (365 nm) at a dose of 13 J/cm^3^ and incubated in 3D system with the standard growing medium for 24 h; **UVA + *N.o*.** group, the fibroblasts exposed to UVA (365 nm) at a dose of 13 J/cm^3^ and then incubated in 3D system with the standard growing medium containing lipid extracts from *N. oceanica* (3 µg/mL) for 24 h; **UVA + *C.a.*** group, the fibroblasts exposed to UVA (365 nm) at a dose of 13 J/cm^3^ and then incubated in 3D system with the standard growing medium containing lipid extracts from *C. amblystomatis* (3 µg/mL) for 24 h]. The relevant protein band has been shown with a black arrow. All the original photos of the membranes are presented in Supplementary Figure S2.

To verify the most significant changes in protein expression observed in the proteomic data, the levels of randomly selected proteins (cytochrome C, light chain ferritin, RAS, peroxiredoxin-5, heat shock protein 10 kDa 1, matrix metalloproteinase 1, and fibulin 1) were determined by Western blotting (Figure 7 and Supplementary Figure S2). In the case of fibroblasts, the bands indicating light chain ferritin and peroxiredoxin-5 showed similar intensity in the **UVA** and **UVA + *C.a.*** groups vs. high intensity in the **CTR group**, similarly to what was observed in the heat map (Figure 3). Furthermore, the intensity of the band corresponding to mitochondrial heat shock protein 10 kDa was higher in the **UVA** and **UVA + *C.a.*** groups vs. **CTR** and ***C.a.*** Although Western blot analysis does not offer the sensitivity of mass spectrometry, it showed that *C. amblystomatis* lipid extract significantly influenced and regulated the expression of redox balance-regulating proteins, including peroxiredoxin-5, cytochrome c, ferritin light chain, and 10 kDa heat shock protein 1 in fibroblasts.

In contrast, the application of *N. oceanica* caused the band indicating the light chain of ferritin in the ***N.o.*** and **UVA + *N.o.*** groups to be less intense than in the **CTR** and **UVA** groups. Moreover, the band of matrix metalloproteinase 1 was found to be the strongest in the **UVA *+ N.o*** group vs. the **CTR**, ***N.o.*** and **UVA** groups (Figure 3). It was also observed that the bands corresponding to fibulin-1 were more visible in the case of *N. oceanica* extract-treated cell groups (***N.o.*** and **UVA *+ N.o***). The extract of *N. oceanica* was found to change the expression of proteins associated with inflammation, such as matrix metalloproteinase-1, fibulin-1 and Ras, as well as proteins related to redox balance, including cytochrome c and ferritin light chain.

**Figure 8:**
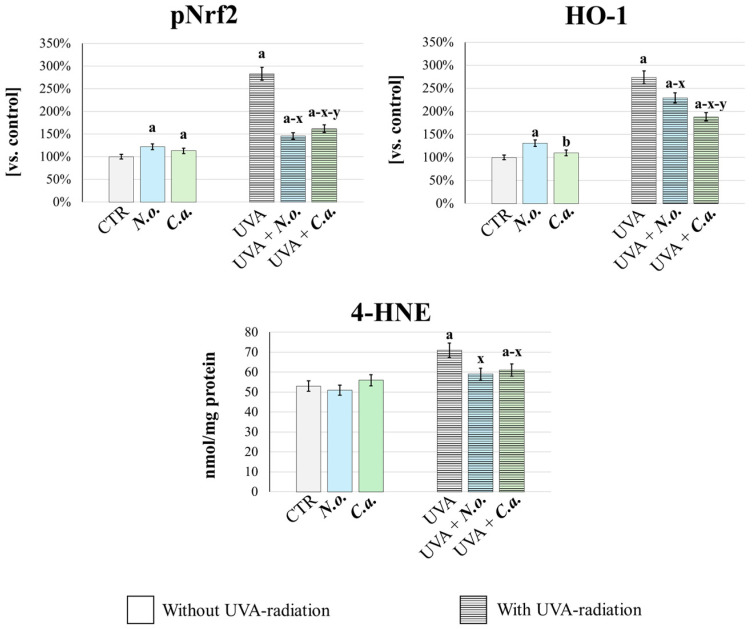
Assessment of cellular antioxidant response and oxidative stress. The level of phosphorylated-Nrf2 (pNrf2, active), heme oxygenase-1 (HO-1) and 4-HNE (lipid peroxidation product, oxidative stress marker) were determinated in fibroblasts [**CTR group**, control fibroblasts cultured in 3D system with standard medium; ***N.o.*** group, the fibroblasts treated with lipid extract obtained from *N. oceanica* (3 µg/mL) for 24 h; ***C.a.*** group, the fibroblasts treated with lipid extract obtained from *C. amblystomatis* (3 µg/mL) for 24 h; **UVA** group, the fibroblasts exposed to UVA (365 nm) at a dose of 13 J/cm^3^ and incubated in 3D system with the standard growing medium for 24 h; **UVA + *N.o*.** group, the fibroblasts exposed to UVA (365 nm) at a dose of 13 J/cm^3^ and then incubated in 3D system with the standard growing medium containing lipid extracts from *N. oceanica* (3 µg/mL) for 24 h; **UVA + *C.a.*** group, the fibroblasts exposed to UVA (365 nm) at a dose of 13 J/cm^3^ and then incubated in 3D system with the standard growing medium containing lipid extracts from *C. amblystomatis* (3 µg/mL) for 24 h]. The levels of pNrf2 and HO-1 (ng/mg protein) were expressed as a percentage of the expression observed in the control cells (as explained in Section 2.6.2) and obtained 4-HNE values were expressed as nmol/mg protein (as explained in Section 2.7). Mean values ± SD of three independent samples and statistically significant differences for *p* ≤ 0.05 are presented: (**a**) is used for differences vs. **CTR**; (**b**) is used for differences between ***N.o.*** group and ***C.a.*** group; (**x**) is used for differences between **UVA** group and **UVA + *N.o*.** group/**UVA + *C.a.*** group; (**y**) is used for differences between **UVA + *N.o*.** group and **UVA + *C.a.*** group.

In order to quantitatively confirm the changes in the redox balance in fibroblasts exposed to UVA or/and algal extracts, the cellular antioxidant response was analyzed (Figure 8). This was assessed by measuring the basal level of the transcription factor responsible for the expression of antioxidant proteins—pNrf2, and its product of efficacy, heme oxygenase-1 (HO-1) [44]. Additionally, oxidative stress intensity was evaluated by determining the level of 4-hydroxynonenal (4-HNE), a well-established marker of lipid peroxidation and oxidative stress [45]. It was found that UVA radiation caused oxidative stress, as evidenced by a significant increase in the generation of 4-HNE. This was accompanied by an increase in the cellular antioxidant and cytoprotective response, as evidenced by a significant increase in the levels of pNrf2 and HO-1. Both lipid extracts, applied after UVA irradiation, effectively reversed these changes: they reduced oxidative stress and the associated antioxidant response as evidenced by the reduction in 4-HNE, pNrf2 and HO-1 levels. Moreover, the lipid extract from *N. oceanica* was more effective in reducing pNrf2 and 4-HNE levels, while the lipid extract from *C. amblystomatis* showed greater efficacy in reducing HO-1 level compared to *N. oceanica*. Importantly, under conditions without UVA radiation, both extracts enhanced the cellular antioxidant response (evidenced by increased levels of pNrf2 and HO-1, compared to the control group) while not inducing oxidative stress, as indicated by unchanged 4-HNE levels vs. CTR group.

**Figure 9:**
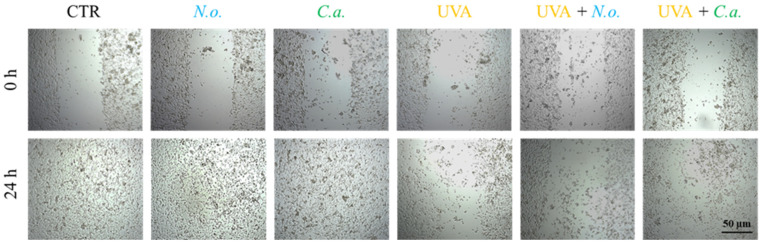
Scratch wound healing of skin fibroblasts treated with *N. oceanica* or *C. amblystomatis* lipid extracts (3 µg/mL) following exposure to UVA (365 nm) at a dose of 13 J/cm^3^.

The obtained results show that UVA radiation significantly reduces the fibroblast proliferation and migration, leading to a decrease in the regenerative capacity of skin. Both of the microalgae extracts used slightly counteracted this effect caused by UVA (Figure 9). In the case of fibroblasts not exposed to UVA radiation, no changes in the growth rate induced by lipid extracts were observed; within 24 h of incubation, the fibroblasts from all un-UVA-exposed groups were able to cover the entire surface of the furrow.

## 4. Discussion

Our study highlights lipid extracts of *N. oceanica* and *C. amblystomatis* as promising candidates for therapeutic interventions targeting oxidative stress and inflammation. These lipid extracts effectively reverse the proteomic alterations in skin fibroblasts induced by UVA irradiation, including those related to critical biological activities such as redox regulation, inflammation, and cell survival in 3D-cultured fibroblasts, closely resembling the native skin fibroblast layer.

The results of the present study indicate differential proteomic–metabolic responses of fibroblasts under the influence of extracts from two different types of algae. Considering the differences in the lipid composition of these extracts [9,10,46,47], it can be suggested that they are significant enough to cause a differential proteomic response.

### 4.1. Potential Cytoprotective Action of C. amblystomatis Lipid Extract: Regulation of Redox Balance and Associated Inflammatory Signaling

Notably, the significant increase in mitochondrial cytochrome c oxidase subunit 4 isoform 1 (CCO4-1) expression induced by UVA was markedly reduced following treatment with both lipid extracts, with *C. amblystomatis* lipid extract demonstrating greater efficacy in reversing this increase. It is well established that stress-induced cytosolic calcium elevation, dephosphorylation of CCO, and the loss of “allosteric ATP inhibition of CCO” contribute to increased mitochondrial membrane potential, ROS production, and potentially apoptosis or disease progression [48]. *C. amblystomatis* lipid extract treatment also counteracted the UVA-reduced expression of another mitochondrial antioxidant protein, peroxiredoxin-5 [49]. These findings underscore the ability of both lipid extracts to restore redox balance disrupted by UVA radiation, with the *C. amblystomatis* lipid extract showing a more pronounced effect by enhancing the suppressed cellular antioxidant response.

**Figure 10:**
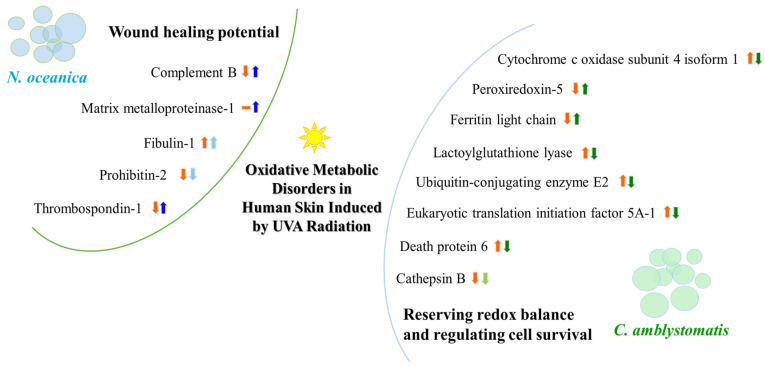
A summary illustration of the wound healing potential of *N. oceanica* lipid extract and the redox balance and cell survival regulatory actions of *C. amblystomatis* lipid extract. The figure highlights alterations in protein expression and their impact on associated intracellular molecular signaling pathways. [An upward arrow signifies an increase, while a downward arrow indicates a decrease in protein expression, within orange color (showing UVA effect) or blue/green color (showing the effect of treatment with *N. oceanica* or *C. amblystomatis* lipid extracts following UVA radiation). Light blue/green colors are used to express the slowing down effect. The dash symbol is used to represent no protein identification.].

*C. amblystomatis* lipid extract reduced the expression of the ferritin heavy chain but increased the level of the light chain of this protein. The ferritin light chain plays an antioxidant role by reducing free iron concentrations through the formation of ferritin complexes with the heavy chain, thereby mitigating the Fenton reaction [50]. The observed increase in ferritin light chain expression following *C. amblystomatis* lipid extract treatment confirms its antioxidant and protective potential (Figure 10).

When using *C. amblystomatis* lipid extract, two notable changes in protein expression were observed: the increased levels of lactoylglutathione lyase and ubiquitin-conjugating enzyme E2 variant 1 caused by UVA exposure were significantly reduced following treatment. Lactoylglutathione lyase (glyoxalase I) is a key enzyme in the metabolism of 2-oxoaldehydes, particularly methylglyoxal (MGO). MGO is detoxified within the glyoxalase system using glutathione (GSH) as a cofactor [51]. Its levels are elevated under oxidative stress [51], and impaired MGO metabolism has been linked to increased oxidative stress and chronic inflammation [51,52]. These findings suggest that, beyond its cytoprotective effects, *C. amblystomatis* lipid extract may protect fibroblasts from chronic inflammation by restoring the UVA-disrupted glyoxalase pathway.

Ubiquitin-conjugating enzyme E2 (UBE2), participating in various skin pathologies including melanoma [53], plays a pivotal role in inflammation by promoting nuclear factor kappa B (NF-κB) activation, pro-IL-1β ubiquitination and its proteasomal degradation [54]. Our findings suggest that *C. amblystomatis* lipid extract indirectly regulates inflammation by altering UBE2 expression. Moreover, UBE2(O) expression is known to increase during wound healing, promoting angiogenesis [55]. Consequently, a reduction in UVA-induced expressions of both glyoxalase I and UBE2 by *C. amblystomatis* lipid extract suggests its role in modulating the pro-inflammatory response triggered by UVA.

This inflammation-regulating effect of *C. amblystomatis* lipid extract is further supported by changes in the expression of prostaglandin E synthase 3, a key lipid mediator with diverse inflammation-regulatory functions [56]. *C. amblystomatis* lipid extract slightly increased the already elevated levels of prostaglandin E synthase 3 caused by UVA exposure. This mild increase could be interpreted as a deceleration in the UVA-induced escalation of this enzyme’s expression, reflecting a nuanced regulatory effect on inflammatory signaling. Beyond that, these findings highlight *C. amblystomatis*’s potential to modulate inflammation. Unlike the 2D study, its effect on inflammation is more pronounced here, alongside its significant role in redox modulation.

*C. amblystomatis* effectively disrupts the dynamics of cell survival by reducing the levels of eukaryotic translation initiation factor 5A-1 (eIF5A1), whose expression increases under UVA radiation. Overexpression of eIF5A1 has been observed in numerous cancers, where it can act as either an oncogene or a tumor suppressor [57]. Reduced eIF5A expression has been linked to mitochondrial fission, a key process in metabolic reprogramming [58], ROS production, and apoptosis [59], which has shown antitumor effects in human ovarian epithelial cancer cell lines [60]. Both lipid extracts, particularly *C. amblystomatis* under the given treatment conditions, hold significant potential for protecting skin against UVA-induced cell proliferation and malignant transformation, through the modulation of eIF5A-mediated apoptosis and ferroptosis.

The application of *C. amblystomatis* lipid extract following UVA exposure of fibroblasts results in a significant decrease in the expression of programmed cell death protein 6 (PDCD6) compared to untreated UVA-irradiated cells. PDCD6 accumulates in the nucleus and participates in apoptosis triggered by DNA damage, mediated by the p53 transcription factor—a tumor suppressor whose expression is upregulated in response to UV radiation [61]. These findings suggest that *C. amblystomatis* lipid extract can restore the disrupted dynamics of cell survival caused by UVA exposure, potentially improving cellular homeostasis. Additionally, the dramatic reduction in cathepsin B levels induced by UVA irradiation appears to be mitigated by *C. amblystomatis* lipid extract treatment. Cathepsin B, a thiol-dependent cysteine protease, is known to be inactivated by UVA, leading to dysfunction of autophagic-lysosomal processes in fibroblasts [62]. Thus, the use of *C. amblystomatis* may attenuate the impairment of cellular protein degradation by preserving lysosomal functionality in UVA-exposed fibroblasts.

Overall, the results of this study are consistent in the direction of changes with previous findings from UVA-irradiated fibroblasts cultured in a traditional 2D model [17], but offers a broader interpretative framework, as detailed above. The higher antioxidant efficiency of *C. amblystomatis* in restoring the redox balance disturbed by UVA radiation is obvious and supported by data on the 2D model [17]. Treatment with *C. amblystomatis* after UVA exposure reduced HO-1 expression, consistent with the results observed in the present experiment. Changes in oxidative stress, as indicated by 4-HNE levels, may rapidly enhance the antioxidant response through a dynamic process involving multiple redox-regulating proteins, including the transcription factor Nrf2 and its product of efficacy, HO-1. Since this treatment also reduced the increase in UVA-induced 4-HNE levels, this may indicate effective mitigation of UVA-mediated oxidative stress. Taken together, these results support the redox-regulating function of this extract. Moreover, it was also noted that the expression of peroxiredoxin 5 increased with *C. amblystomatis* treatment even in the absence of UVA exposure.

In the previous 2D study, UVA-disrupted expression of two additional antioxidant enzymes, aldo-keto reductase family member 1A1 and thioredoxin, is also found to be restored by *C. amblystomatis* treatment following UVA exposure [17]. Redox signaling modulation is a highly dynamic molecular process involving the participation of numerous antioxidant proteins [63]. Consequently, variations in experimental conditions and the time-dependent dynamic responses of cells account for the differences observed in antioxidant protein expression. However, these findings by demonstrating the modulatory effect of *C. amblystomatis* on redox regulation in fibroblasts further highlight the key role of peroxiredoxin 5 expression in the regulatory effect of this extract.

Together with this, another study on skin fibroblasts highlights a reduction in ROS levels induced by UVA, an increase in Nrf2 expression, and enhanced activity/levels of antioxidants, including thioredoxin, due to *C. amblystomatis* lipid extract treatment [16]. The mass spectrometry used in this study is characterized by higher sensitivity compared to the ELISA method used in the mentioned study [16]. Moreover, the cell culture methodology used in the two studies is different, 3D and 2D [16] cell culturing. Considering these differences, dynamic changes in antioxidant protein expression may also be a result of the concentration used and/or time-dependent changes in cellular response, which requires further evaluation. However, this extract demonstrates significant potential in restoring intracellular redox equilibrium disrupted by UVA irradiation. This potential is further supported by a recent proteomic study on 3D-cultured melanoma cells, which demonstrated the reducing effect of *C. amblystomatis* on the UVA-induced increase in 4-HNE-protein adducts in both cells and the surrounding medium [47]. Our 3D fibroblast results support these findings, showing that treatment with *C. amblystomatis* lipid extract after UVA radiation reduced the UVA-induced increase in 4-HNE generation. In addition, the ELISA and GC-MS results obtained in this study validate the 3D-proteomic findings and further highlight the antioxidant role of this extract, as it counteracts UVA-induced oxidative stress and the associated antioxidant response by significantly reducing UVA-induced 4-HNE generation, Nrf2 activation, and HO-1 expression. On the other hand, the enrichment of cytoplasmic translation and ribosome biogenesis in the case of *C. amblystomatis* lipid extract may suggest the role of this extract in promoting protein production [64], and cellular adaptation to UVA-induced stress [65].

Unlike our previous study [17], the IP-adjusted proteome analysis in this study identified a greater number of proteins, making it statistically more robust. The treatment with *C. amblystomatis* lipid extract significantly reduced the UVA-induced increase in mitochondrial superoxide dismutase [Mn] (SOD2) levels within the caspase-1 interactome. While the regulatory role of SOD1 in caspase-1 activity has been demonstrated using SOD1-deficient macrophages and mice [66], our findings highlight that SOD2 may similarly influence caspase-1 activation.

*C. amblystomatis* lipid extract treatment may mitigate the pro-inflammatory response triggered by UVA by negatively regulating caspase-1 activity. Furthermore, the significant decrease in SOD2 expression observed in the IP-adjusted analysis underscores the crucial role of the *C. amblystomatis* extract in restoring the redox imbalance caused by UVA exposure, as highlighted earlier through the complete proteomic analysis. *C. amblystomatis* lipid extract may support the modulation of inflammation by regulating caspase-1 signaling (and potentially inflammasome activity, indirectly) while restoring the redox balance disrupted by UVA.

### 4.2. Potential Effects of N. oceanica Lipid Extract Treatment on the Wound Healing Process, as Indicated by Changes in the Protein Profile of Fibroblasts

Following *N. oceanica* lipid extract treatment, the UVA-decreased expression of complement factor B was restored. And interstitial collagenase (matrix metalloproteinase-1, MMP1) was detected with high intensity only in UVA-irradiated fibroblasts treated with N. oceanica lipid extract. The complement system is a key player in innate immune defense and inflammation [67], with its elements modulated by metabolic and oxidative stress to ensure a proper cellular response [68]. The complement factor B, a serine protease, catalyzes complement activation and the initiation of inflammation [69]. Matrix metalloproteinases are critical for restoring damaged skin and facilitating wound healing [70]. Complement activation also contributes to tissue repair during the inflammation phase of wound healing [71]. The *N. oceanica* lipid extract-mediated increase in complement factor B and MMP1 levels may reflect its modulation of inflammatory signaling during the wound healing process. This conclusion is further supported by the increased expression of plasma serine protease inhibitor and thrombospondin-1, both of which have pro-inflammatory roles in wound healing [72,73,74], following *N. oceanica* lipid extract treatment after UVA exposure.

The cells treated with the lipid extract of *N. oceanica* displayed increased levels of fibulin-1, an extracellular matrix protein involved in matrix organization, cell proliferation, migration, and wound healing in vascular smooth muscle cells [75]. While fibulin-1 levels are not reduced in sun-exposed reticular dermis of young, sun-protected skin [76], UVA exposure decreases fibulin-5 levels, and fibulin-7 overexpression suppresses keratinocyte differentiation and slows proliferation [77]. Thus, the observed increase in fibulin-1 levels following *N. oceanica* lipid extract treatment may reflect its involvement in the wound healing process, although further analysis is necessary, particularly given the increase observed in control cells without UVA exposure.

One of the protective mechanisms for cells—sensitive to oxidative stress—is mitophagy acting through prohibitin-2, which reduces apoptosis via the Nrf2/prohibitin-2 pathway [78]. In this study, prohibitin-2 levels were dramatically reduced by UVA exposure, but treatment with *N. oceanica* lipid extract slowed this decline. This may suggest that the extract reduces UVA-induced oxidative stress and supports cell survival during the healing process. This protective effect is further corroborated by the increased expression of thrombospondin-1, a high-intensity protein identified only in cells exposed to UVA radiation and treated with lipid extract from *N. oceanica*. Thrombospondin-1, a cell matrix protein involved in tissue remodeling and wound healing, facilitates epithelial cell migration in response to injury [79].

A previous 2D study showed that *N. oceanica* treatment significantly increases MMP1 expression following UVA irradiation in 2D-cultured fibroblasts [17]. The increase in plasma serine protease inhibitor and thrombospondin-1 observed in this study further strengthens the potential of this lipid extract to promote wound healing. Also, the previous 2D study shows that *N. oceanica* treatment increases the expression of heme oxygenase-1 in UVA-treated fibroblasts [17]. HO-1, the enzyme in heme degradation, is involved in wound repair and resolution of inflammatory response [80]. In addition, a recent study in melanoma cells indicates a significant increase in the same extract’s effect on UVA-decreased placenta growth factor (PGF) and vascular endothelial growth factor (VEGF) expression [47]. The mesoglycan/VEGF interaction is suggested to promote new vessel formation and granulation tissue deposition by stimulating endothelial and fibroblast cell components in skin wound healing [81]. Notably, PGF is also upregulated during wound healing, promoting angiogenesis during skin repair [82]. This is further supported by the delayed wound closure observed in PGF-deficient mice [82].

Furthermore, the melanoma study also indicated significant protein expression variations in the medium [47]. The most prominent proteins detected included epidermal growth factor receptor (EGFR), protein disulfide-isomerase A4 (PDIA4), fibroblast growth factor 2 (FGF2), and pro-transforming growth factor β3 (TGF β3). The mentioned proteins are involved in the wound healing process [83,84]/platelet activation and thrombosis formation [85]/tissue repair and regeneration [86]. Also, *N. oceanica* lipid extract caused more significant changes in the expression of the proteins participating in inflammatory signaling in melanoma cells, versus *C. amblystomatis* [47].

Moreover, another study focusing on the changes in intracellular lipid metabolism highlights a decrease in eicosanoids, such as prostaglandin E2 (PGE2), 15-deoxy-delta-12,14-prostaglandin J2 (15d-PGJ2), thromboxane B2 (TXB2), and 15-hydroxyeicosatetraenoic acid (15-HETE), whose expression increases after UVA radiation in skin fibroblasts [16]. These important lipid mediators which are produced in a time-dependent manner after injury can critically regulate wound healing, positively or negatively [87]. Therefore, this may serve as an important indicator of metabolic reprogramming in skin wound healing, driven by dynamic changes in lipid metabolism [88]. Both lipid mediators mentioned here contribute to different stages of the healing process through their dynamically changed production levels and their influence on inflammation [87,89].

Additionally, earlier work highlights the restorative effects of *N. oceanica* lipid extract on phospholipid metabolism in UVB-irradiated keratinocytes [13]. Our proteomics study provides insight into evaluating the wound healing potential of *N. oceanica* lipid extract. However, future functional experiments are needed to assess this potential in disease models where UV radiation plays a critical role, such as sunburn, actinic keratosis, and skin cancers [90,91]. Therefore, the potential of *N. oceanica* lipid extract to accelerate wound healing requires further investigation (Figure 7).

This 3D study also reveals that both lipid extracts significantly decrease ribosomal protein expression, in general, with this effect being exacerbated by UVA exposure. Interestingly, this result contradicts the findings from our previous study [17]. Generally, oxidative stress reduces translation and damages mRNA and ribosomal proteins [92], while redox signaling dynamically regulates the chaperone-mediated ribosome repair mechanism. Moreover, UV radiation, by damaging RNA, induces not only oxidative stress but also ribotoxic stress [93]. A recent study highlights that the immediate-early response to UV exposure is dominated by ribosome-mediated signaling, with UV-induced apoptosis being driven by the ribotoxic stress response rather than the DNA damage response [94]. The observed differences in ribosomal protein expression between the studies may be attributed to the 3D culture model, which better replicates complex and dynamic cellular responses [18]. Additionally, its multilayer structure may cause variations in oxidative stress intensity, timing, and localization due to differences in physicochemical factor diffusion and cell susceptibility across layers. Furthermore, the dynamics of ribotoxic stress, whose regulatory mechanisms remain poorly understood, may also play a significant role. It is plausible that the molecular activity of the lipid extracts extends to regulating ribotoxic stress associated with UV radiation.

The (GO) enrichment data provide further insights into the mechanism of actions of *N. oceanica* lipid extract. The positive regulation of protein processing in phagocytic vesicles as well as fibrinolysis may suggest the role of *N. oceanica* in altering UVA response by influencing protein processing, immune response, and inflammation resolution [95], and/or fibrin breakdown, aiding tissue repair and wound healing [96].

Fibroblast migration and proliferation of fibroblasts are essential elements of the healing process, as they trigger the proliferative phase of tissue repair [97]. The results of the wound healing analysis indicate that both lipid extracts can slightly counteract the effects of UVA by reducing fibroblast proliferation and migration. However, the significant difference in the effects indicated at the proteome level, especially in the case of *N. oceanica*, may be due to the time differences between protein expression and the response seen in the cellular phenotype.

Our data also highlight the regulatory potential of *N. oceanica* extract in reducing UVA-mediated oxidative stress. This finding is particularly significant considering the critical role of redox signaling in the wound healing process [3].

For *N. oceanica* lipid extract treatment within the IP-adjusted proteome analysis, the expression levels of three proteins—actin (cytoplasmic 2), beta-globin protein, and transthyretin—were reduced following UVA exposure. Neutrophil elastase binds to and degrades F-actin, disrupting actin dynamics, and it translocates to the nucleus, where it degrades histones and facilitates chromatin decondensation. At the same time, the pore-forming fragment of Gasdermin D (N-GSDMD), generated by caspase-1, directs azurophilic granules to release neutrophil elastase into the cytosol [98,99,100]. While no direct connection between beta-globin and caspase-1 has been established, studies indicate that heme—a component of hemoglobin including the beta-globin subunit [101]—can promote inflammation via NLRP3 inflammasome activation and subsequent caspase-1 activity [102,103]. Moreover, transthyretin oligomeric and fibrillar species have been shown to activate NF-κB signaling, leading to the expression of inflammation-associated molecules such as pro-inflammatory cytokines (e.g., IL-1β and IL-18) and the components of inflammasome complexes (e.g., NLRP3) [104,105]. Although the direct interactions of these proteins with caspase-1 remain unreported in the literature, our findings suggest that *N. oceanica* lipid extract may modulate the UVA-induced inflammatory response.

### 4.3. Limitation of the Study

The present proteomic analysis provides new data on the biological activity of algal lipid extracts, including the potential redox regulatory role of *C. amblystomatis* extract and the effect of *N. oceanica* extract on wound healing. However, the lack of detailed functional validation is a key limitation of this study. Therefore, further studies are needed to determine the measurable significance of the obtained results, including transwell migration and invasion assays, as well as analysis of in vivo wound healing models to determine the causal relationship between the proteomic results and functional changes. Furthermore, the present results indicating changes in the protein profile of the caspase-1 environment do not indicate specific protein–protein interactions and consequent structural–functional modifications. However, the unexplained elements indicated in this study will become a starting point for further evaluation of proteome modifications under the influence of algal extracts.

## 5. Conclusions

Lipid extracts from various species of microalgae differentially modify the proteomic profile of fibroblasts by influencing different metabolic pathways. *C. amblystomatis* lipid extract alters the profile of proteins involved in intracellular redox signaling by reversing UVA-induced changes in the expression of mitochondrial cytochrome c oxidase, peroxiredoxin-5 and ferritin, and proteins cooperating in the chronic inflammatory response (Mn-SOD—caspase 1). In contrast, the wound healing potential of *N. oceanica* lipid extract is primarily associated with increased levels of complement factor B, MMP1, fibulin-1 and thrombospondin-1. Therefore, from a pharmacotherapeutic perspective, both lipid extracts show promising effects in the metabolic protection of skin cells, each through distinct mechanisms of action.

## Data Availability

The data generated and presented in this study are included in the article and are available in detail as supplementary data. Further inquiries can be directed to the corresponding author.

## Supplementary Materials

The following supporting information can be downloaded at: https://www.mdpi.com/article/10.3390/antiox14050545/s1, Supplementary Figure S1: SDS-PAGE gels in which the samples containing total protein extracts or the IP samples, obtained from the experimental fibroblast groups. Supplementary Figure S2: Western blot analysis against β-actin, cytochrome C, ferritin light chain, RAS, peroxiredoxin 5, heat shock 10 kDa protein 1, matrix metalloproteinases 1 or fibulin 1 in the case of *N. oceanica* or *C. amblystomatis* lipid extract treatment of fibroblasts. Supplementary Table S1: The list of identified proteins within the complete proteomic analysis or IP-adjusted proteomic analysis as well as the list of significantly altered proteins in the case of *N. oceanica* or *C. amblystomatis* lipid extract treatment within the complete proteomic analysis or IP-adjusted proteomic analysis. Supplementary Table S2: Fold-changes between fibroblast groups in the case of *N. oceanica* or *C. amblystomatis* lipid extract treatment within the complete proteomic analysis or IP-adjusted proteomic analysis.
