# Supplementary material for: The Effects of Lipid Extracts from Microalgae Chlorococcum amblystomatis and Nannochloropsis oceanica on the Proteome of 3D-Cultured Fibroblasts Exposed to UVA Radiation
Source: Antioxidants (Basel). 2025 Apr 30;14(5):545. doi: 10.3390/antiox14050545 (PMC12108275; doi:10.3390/antiox14050545)
Supplement: Supplementary file 1 [file antioxidants-14-00545-s001.zip › Supplementary Figures.pdf]

A

B

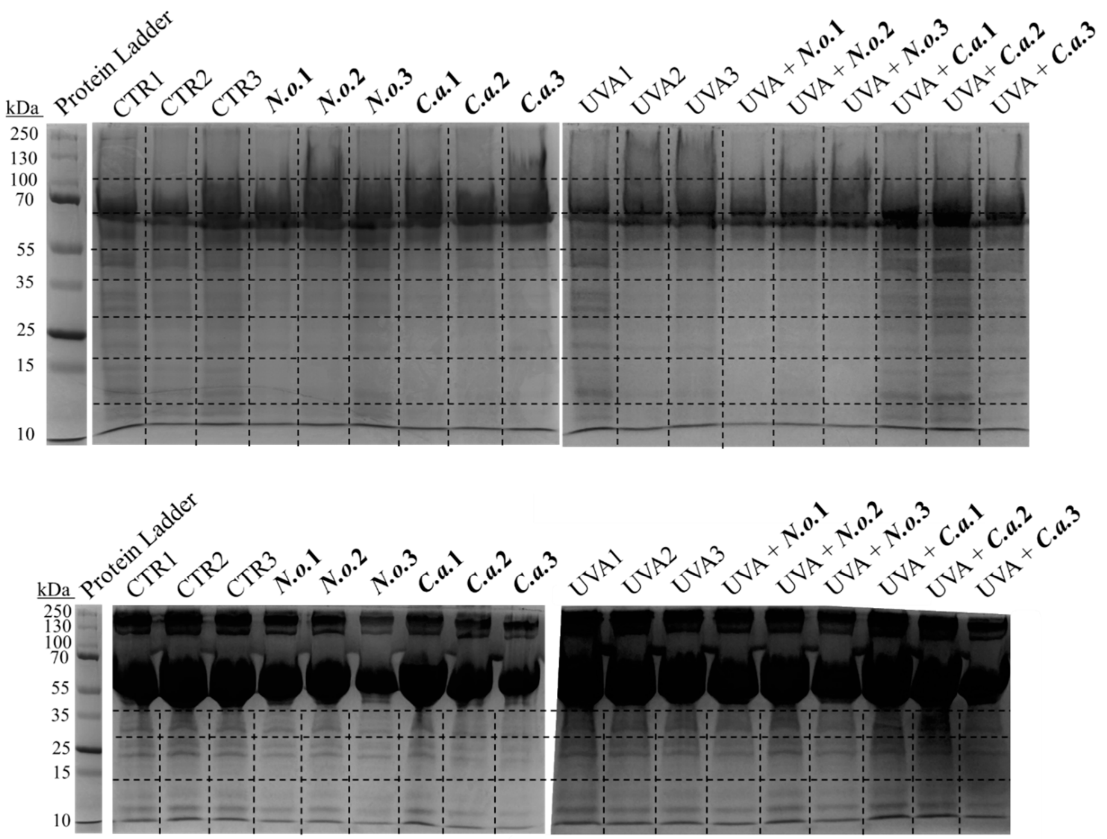

Figure S1. SDS-PAGE gels in which the samples containing total protein extracts (**A**) or the IP samples (**B**), obtained from the experimental fibroblast groups [**CTR 1/2/3** group, control cells cultured in 3D system with standard medium; ***N.o.* 1/2/3** group, the cells treated with lipid extract obtained from *N. oceanica* (3µg/ml) for 24h; ***C.a.* 1/2/3** group, the cells treated with lipid extract obtained from *C. amblystomatis* (3µg/ml) for 24h; **UVA 1/2/3** group, the cells exposed to UVA (365 nm) at a dose of 13 J/cm^3 and incubated in 3D system with the standard growing medium for 24h; **UVA + *N.o.* 1/2/3** group, the cells exposed to UVA (365 nm) at a dose of 13 J/cm^3 and then incubated in 3D system with the standard growing medium containing lipid extracts from *N. oceanica* (3µg/ml) for 24h; **UVA + *C.a.* 1/2/3** group, the cells exposed to UVA (365 nm) at a dose of 13 J/cm^3 and then incubated in 3D system with the standard growing medium containing lipid extracts from *C. amblystomatis* (3µg/ml) for 24h], were separated and then stained by Coomassie brilliant blue. The way of gel cutting-slicing into eight (**A**) or three (**B**) sections for the following in-gel trypsinization step is presented by the white dashed line. For IP samples (**B**), the parts that were not in the dash lines, which were observed very intensively depending on the IP procedure, were discarded and not included in the following proteomic analysis, to avoid dominating proteomic analysis. (**IP**, immunoprecipitation against human caspase-1 used here).

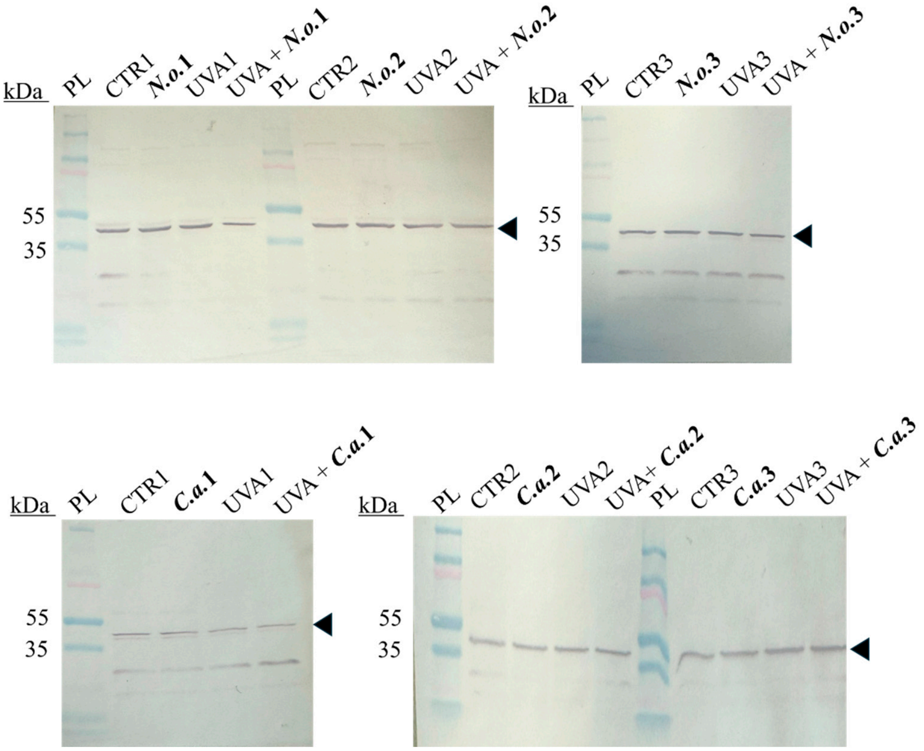

Figure S2. Western blot analysis against β-actin [**CTR 1/2/3** group, control cells cultured in 3D system with standard medium; ***N.o.* 1/2/3** group, the cells treated with lipid extract obtained from *N. oceanica* (3μg/ml) for 24h; ***C.a.* 1/2/3** group, the cells treated with lipid extract obtained from *C. amblystomatis* (3μg/ml) for 24h; **UVA 1/2/3** group, the cells exposed to UVA (365 nm) at a dose of 13 J/cm^3 and incubated in 3D system with the standard growing medium for 24h; **UVA + *N.o.* 1/2/3** group, the cells exposed to UVA (365 nm) at a dose of 13 J/cm^3 and then incubated in 3D system with the standard growing medium containing lipid extracts from *N. oceanica* (3μg/ml) for 24h; **UVA + *C.a.* 1/2/3** group, the cells exposed to UVA (365 nm) at a dose of 13 J/cm^3 and then incubated in 3D system with the standard growing medium containing lipid extracts from *C. amblystomatis* (3μg/ml) for 24h]. The relevant protein band has been shown with black triangle.
